# Editorial: Case reports in cardiovascular imaging: 2022

**DOI:** 10.3389/fcvm.2023.1222166

**Published:** 2023-06-23

**Authors:** Antonios Karanasos, Riccardo Liga, Grigorios Korosoglou

**Affiliations:** ^1^ 1st Department of Cardiology, Athens Medical School, Hippokration Hospital, Athens, Greece; ^2^ Department of Surgical Pathology, University of Pisa, Pisa, Italy; ^3^ Cardiothoracic and Vascular Department, University Hospital of Pisa, Pisa, Italy; ^4^ Department of Cardiology, Vascular Medicine and Pneumology, GRN Hospital Weinheim, Weinheim, Germany; ^5^ Cardiac Imaging Center Weinheim, Hector Foundation, Weinheim, Germany

**Keywords:** echocardiography, cardiac magnetic resonance (CMR), cardiac computed tomography (CCT), multimodal imaging, tissue characterization, cardiac masses, diagnostic classification, positron emission tomography (PET)

**Editorial on the Research Topic** Case reports in cardiovascular imaging: 2022

Technical developments with several cardiovascular imaging techniques, including echocardiography, cardiac magnetic resonance (CMR), cardiac computed tomography angiography (CCTA) and positron emission tomography (PET) contributed to the profound understanding of cardiac physiology, as well as to precise diagnostic classification and risk stratification of patients with different cardiovascular disorders. Together with the wider availability of cardiovascular imaging and continuous training of cardiologists and radiologists in terms of image acquisition, analysis and interpretation within the clinical context, imaging techniques may not only provide the correct diagnosis, but also aid the monitoring of treatment strategies. In addition, artificial intelligence may standardize and harmonize the diagnostic processes, further enhancing the role of cardiovascular imaging in clinical practice. This taken together, along with continuous spread of knowledge among experts from different disciplines, like with the case series presented herein, will contribute to improved diagnostic work-up, patient management and outcomes.

The role of multimodality imaging is highlighted in the article by Yang D. et al. where using contrast echocardiography, CCTA and PET-CT a cardiac lymphoma was suspected. Using this multimodality imaging approach, the localization of the tumor in relation to neighboring structures of the heart could be verified, while extra cardiac spread helped classifying the structure as malignant, providing important information for the subsequent clinical treatment of the patient.

The role of cardiovascular imaging was again highlighted in another interesting case by Punzo et al. where CCTA and CMR aided determination of lesion morphology, anatomical localization, and tissue characterization in a patient with cardiac paraganglioma. Cardiac paragangliomas are rare extra-adrenal tumors, which arise from chromaffin cells of sympathetic ganglia and are frequently diagnosed incidentally in the absence of limiting clinical symptoms. The role of CMR, together with other imaging modalities, in the sense of a multimodal approach for the diagnosis of cardiac tumors has already been highlighted in a recent multi-center study (1) and has been discussed accordingly (2). The above-mentioned cases nicely confirm the importance of multimodality imaging and interdisciplinary discussion in this context.

In another interesting case report, Wa et al. demonstrated the importance of multimodality cardiovascular imaging for the establishment of the correct diagnosis in a relatively rare disease, cardiac sarcoidosis. Hereby, the presence of late gadolinium enhancement (LGE) in the right ventricle by CMR was indicative of sarcoidosis, whereas PET confirmed intense cardiac uptake in the septum and in the right ventricle. Imaging was used to guide endomyocardial biopsy, which indeed confirmed the presence of epithelioid nodules and lymphocytes indicative for cardiac sarcoidosis. Thereafter, the patient received immunosuppressive treatment with prednisolone, which led to resolution of the limiting symptoms and regression of the original lesion by PET.

In the same direction, Sharifkazemi et al. reported on 2 patients with large coronary artery fistulas, one from the left circumflex artery to the coronary sinus and one to the superior caval vein, which were both diagnosed using transesophageal echocardiography, CCTA and coronary angiography. This combined assessment by multimodality cardiovascular imaging helped to establish the diagnosis and estimate the exact course of the artery fistulas, which may help to predict the disease course and select the most suitable treatment option for the individual patient.

Another interesting case report by Nguyen et al. underlines the role of integration of clinical, multimodal imaging and histologic data for the diagnosis of concomitant intramyocardial and intrahepatic hydatic cysts in a young female patient with echinococcosis. The initial abdominal ultrasound revealed a large cyst in the liver, whereas echocardiography detected a cyst within lateral wall of the left ventricle. CCTA and CMR helped to localize and define typical features of the intramyocardial cyst, which was then treated by cardiac surgery and adjunctive pharmacotherapy.

Another promising development in the section of cardiovascular imaging is the increasing use of point-of-care ultrasound imaging for decision making in acute settings. Ngoc Le et al. were able to detect two large heterogeneous masses in the left ventricular wall and in the apical myocardium of a patients presenting with suspected acute ST-elevation myocardial infarction. This completely changed the initial diagnosis, modifying the treatment strategy in a patient with metastatic oesophageal carcinoma, where subsequent CCTA confirmed the presence of multiple cardiac and lung metastases.

In addition, imaging can shed light to characteristic of cardiac or vascular diseases, which are not completely understood in terms of pathology and pathophysiology. Thus, based on the report by Hong et al. a case of focal renal artery fibromuscular dysplasia was detected, which gradually progressed to a branch of the main renal artery 8 years after the initial treatment with a bare metal stent. Intravascular imaging has been used to evaluate vascular pathology in the renal arteries (3), and in this case excluded neointimal hyperplasia within the old stent and confirmed the presence of *de novo* eccentric intimal thickening, causing eccentric stenosis in the branch renal artery.

Given the wide epidemiology of inflammatory heart diseases and the relevant central role of cardiovascular imaging, six articles of the collection involved such cases, five of which involved valvular endocarditis. Interestingly, two papers reported cases of Libman-Sacks (LS) endocarditis (Liang et al.; Bui et al.), a form of non-bacterial thrombotic endocarditis (NBTE) that may develop in the presence of a hypercoagulable state, such as antiphospholipid antibody syndrome (APS) (4). Another case by Miao et al. highlighted the relevance of advanced non-invasive cardiac imaging techniques for the characterization of complex patients with blood culture-negative endocarditis, in whom novel innovations in three-dimensional echocardiography provided photorealistic images of cardiac structures, enabling finer anatomical characterization of the disease burden. Two last reports described the fate of patients with bacterial endocarditis caused by “conventional” germs from the streptococcus and staphylococcus family in patients with predisposing condition, such as a congenital heart disease (Yang G. et al.) and an implanted valvular prosthesis (Wu et al.). Both cases demonstrated how a detailed imaging assessment may allow a proper characterization of the endocarditis and of its complications, guiding the subsequent patient management.

Moving from valvular bacterial to autoimmune eosinophilic endocarditis, a paper by Li Y. et al. reported the characteristics and clinical course of the relatively rare Loeffler disease in a patient with multisystemic involvement of eosinophilic granulomatosis with polyangiitis (EGPA), where state-of-the-art cardiac magnetic resonance imaging allowed an in-depth depiction of disease progression.

Seven articles, on the other hand, dealt with cases of congenital heart diseases (CHD), each outlining the importance of a multiparametric imaging assessment to guide both diagnosis and patient management. Two papers described rare congenital anomalies involving the atrial chambers. In one case by Fukudome et al. the management of a patients with a very rare bronchogenic cyst in the right atrium was reported, describing the typical imaging features of the tumor and its preferential management strategy. In a second article, Li R. et al. reported on the clinical presentation, multimodality imaging assessment and final management strategy of a patient with a giant left atrial appendage aneurysm (LAAA). Another rare CHD with multi-chamber cardiac involvement was reported by Ge et al. In their article, a fatal case of double-chambered right ventricle associated with hypertrophic cardiomyopathy and incessant atrial flutter was described, and the specific role of the different cardiac imaging modalities was further discussed in this context. The possible risks of non-cardiac surgery in patients with unknown complex vascular malformations are outlined in the article by Zhang et al. reporting the 8th known case of azygos continuation of the inferior vena cava in a patient undergoing esophagectomy, indicating the benefit of a thorough pre-procedural vascular evaluation to avoid possibly fatal post-procedural complications.

In three articles of the current collection, non-invasive imaging identified the presence of congenital heart conditions that had been asymptomatic until late in the adulthood. The case by Deng et al. describes the finding of double orifice mitral valve by echocardiography. Despite its impressive visual appearance, this anomaly does not require a specific treatment unless combined with significant stenosis or insufficiency. In the case of Tang et al. a simple chest radiograph performed for non-specific symptoms led to the discovery of the rare finding of supracardiac total anomalous pulmonary venous return, which was combined with a large atrial septal defect (ASD). The latter was assessed by a combination of TTE, CCTA, and cardiac catheterization, confirming the functional significance of the disorder and guided successful surgical correction. Finally, the case of Ma et al. illustrated how CCTA and TTE provided incremental information to the invasive angiogram for the diagnosis of a rare case of STEMI with cardiogenic shock in an acute setting. This condition was attributed to the rupture of a giant right sinus of Valsalva aneurysm, causing right coronary artery compression. Despite emergent surgical treatment the outcome was unfavourable in this patient, indicating the severity and poor prognosis of such a condition.

The use of imaging for the identification and management of complications following cardiac interventions is demonstrated in two articles of the collection. The cases presented by Zhou et al. underscore the need for vigilance and echocardiographic follow-up post neochord implantation. Two patients experienced recurrent dyspnoea due to early-intermediate rupture of neochords and were managed by elective reoperation. In another series by Wang et al. the management of the dislodgement of left atrial appendage closure (LAAC) devices is described. The authors used a combination of echocardiography and CCTA to identify the migration of two different devices used for LAAC, and managed to successfully retrieve them percutaneously through the femoral artery in one case and the femoral vein in the other case. Both interventions for complication management were guided using transoesophageal echocardiography.

The novel concept of using three-dimensional printing for procedural planning is illustrated in the article of Carrabba et al. In this interesting case, the authors used CCTA to reconstruct and print a 3d model of the left ventricle (LV) in a patient with ischemic LV dysfunction and LV aneurysm, scheduled for CABG and aneurysmatectomy. The printed model enhanced the 3D conceptualization of the procedure, leading to an improved LV function on this patient, with good agreement of the predicted and surgically attained residual LV volumes. Of course, the clinical value of this promising strategy still merits further investigation in future clinical trials.

In two of the articles of the collection, specific potential issues of imaging are addressed. Liao et al. presented a rare case of contrast-induced encephalopathy (CIE) with extreme manifestations due to contrast administered during a percutaneous coronary intervention. Further neuroimaging studies by brain CT, brain MRI and MRA disclosed the presence of moyamoya disease that in combination with electrolyte and thyroid function abnormalities were speculated to play a role in the severity and duration of the encephalopathy that eventually recovered following a standard treatment regimen for CIE. In the cases by Du et al. the issue of the “watershed” phenomenon in patients with mechanical circulatory support devices is highlighted. The sites of flow mix by cardiac output and mechanical circulatory support devices may be identified by CCTA as an arc-shaped low-attenuation filling defect that needs to be differentially diagnosed by thrombus. In such cases, repeating the examination in a different body position may lead to disappearance of the filling artifact and identification of the “watershed” site.

Finally, two articles illustrate the value of CT angiography in diagnosing vascular pathologies. The article by Ye et al. demonstrates the incremental value of CT angiography for the diagnosis of thrombotic foci in a patient with myocardial infarction due to histologically documented essential thrombocytosis and for guiding the type and duration of additional antithrombotic treatment, while the case by Li X et al. demonstrates the utility of multimodality imaging by colour Doppler ultrasound, CT angiography and digital subtraction angiography in the diagnosis, procedural planning and follow-up of a rare case of abdominopelvic arteriovenous malformation.

In conclusion, recent advances with non-invasive and invasive imaging are immense and decisive in modern cardiovascular medicine. The correct choice and interpretation of cardiovascular images enable precise diagnostic work-up of patients with cardiac diseases, guiding timely and efficient treatment. In addition, intracardiac or transesophageal imaging can effectively guide cardiac interventions during complications, which may occur during coronary or structural heart interventions. Many of the cases reported in our collection are very nice examples, showing how multimodality imaging can be incorporated in the daily clinical practice to improve patient care and resultant outcomes.
